# Pre-clinical evaluation of the MDM2-p53 antagonist RG7388 alone and in combination with chemotherapy in neuroblastoma

**DOI:** 10.18632/oncotarget.3504

**Published:** 2015-03-10

**Authors:** Lindi Chen, Raphaël F. Rousseau, Steven A. Middleton, Gwen L. Nichols, David R. Newell, John Lunec, Deborah A. Tweddle

**Affiliations:** ^1^ Newcastle Cancer Centre, Northern Institute for Cancer Research, Newcastle University, Newcastle, United Kingdom; ^2^ Genentech Inc., South San Francisco, CA, USA; ^3^ Hoffmann-La Roche Inc., Nutley, NJ, USA

**Keywords:** neuroblastoma, MDM2-p53 antagonists, RG7388, combination therapy, Calcusyn

## Abstract

Neuroblastoma is a predominantly p53 wild-type (wt) tumour and MDM2-p53 antagonists offer a novel therapeutic strategy for neuroblastoma patients. RG7388 (Roche) is currently undergoing early phase clinical evaluation in adults. This study assessed the efficacy of RG7388 as a single-agent and in combination with chemotherapies currently used to treat neuroblastoma in a panel of neuroblastoma cell lines. RG7388 GI_50_ concentrations were determined in 21 p53-wt and mutant neuroblastoma cell lines of varying *MYCN*, *MDM2* and *p14^ARF^* status, together with MYCN-regulatable Tet21N cells. The primary determinant of response was the presence of wt p53, and overall there was a >200-fold difference in RG7388 GI_50_ concentrations for p53-wt *versus* mutant cell lines. Tet21N MYCN+ cells were significantly more sensitive to RG7388 compared with MYCN− cells. Using median-effect analysis in 5 p53-wt neuroblastoma cell lines, selected combinations of RG7388 with cisplatin, doxorubicin, topotecan, temozolomide and busulfan were synergistic. Furthermore, combination treatments led to increased apoptosis, as evident by higher caspase-3/7 activity compared to either agent alone. These data show that RG7388 is highly potent against p53-wt neuroblastoma cells, and strongly supports its further evaluation as a novel therapy for patients with high-risk neuroblastoma and wt p53 to potentially improve survival and/or reduce toxicity.

## INTRODUCTION

The p53 protein plays a central role in tumour suppression, by regulating the expression of numerous downstream target genes involved in cellular processes such as apoptosis, cell cycle arrest, differentiation and senescence. Under normal cellular conditions, p53 is maintained at low levels due a tightly regulated negative feedback loop involving the critical negative regulator, MDM2. MDM2 is an E3 ubiquitin ligase, induced in response to p53 activation to directly bind p53 and inhibit its transcriptional activity, as well as promote the nuclear export and targeting of p53 for ubiquitin mediated proteasome degradation. p14^ARF^ is a tumour suppressor and a negative regulator of MDM2 (reviewed by [1]). The importance of p53 in human cancer is emphasised by observations that p53 is mutated in up to half of all malignancies, whilst aberrant upstream or downstream p53 pathways, including *MDM2* amplification and *p14^ARF^* inactivation are common events in p53 wild-type (wt) cancers [2].

MDM2-p53 binding antagonists are a novel class of anti-cancer therapeutics currently in early clinical development, which act by disrupting the interaction between p53 and MDM2 to non-genotoxically activate wt p53. Hoffmann-La Roche were the first to report potent and selective small molecule MDM2-p53 binding antagonists, the *cis*-imidazoline (Nutlin) compound series [3]. To date, Nutlin-3 has been shown to stabilise p53 and activate the p53 pathway, inducing cell cycle arrest, apoptosis, differentiation and/or senescence, in several p53 wt pre-clinical cancer models. The lead *cis*-imidazoline, RG7112, was subsequently the first of its class to enter clinical trials and despite demonstrating proof-of-mechanism in adult *MDM2*-amplified liposarcoma patients [4], results from several Phase I trials indicated highly variable bioavailability, a poor tolerability to daily oral administration and thrombocytopenia as a dose-limiting toxicity [5]. Subsequently, RG7388, a pyrrolidine and second generation MDM2-p53 antagonist from Hoffman-La Roche with enhanced potency, selectivity and bioavailability, and available in both oral and intravenous (IV) formulations has been developed [6]. To overcome tolerability issues with daily administration, intermittent schedules of RG7388, which may enable the bone marrow to recover have advanced to clinical evaluation in adults alone and in combination (www.clinicaltrials.gov; NCT01462175; NCT01773408; NCT02098967) [7, 8]. RG7388 is anticipated to enter paediatric early phase trials in the near future.

Neuroblastoma is an embryonal malignancy of the developing neural crest accounting for 8-10% of all paediatric cancers but 15% of childhood cancer mortality [9]. Over 50% of patients present with high-risk metastatic disease at the time of diagnosis. Despite an initial response to intensive multimodal therapy, relapse with chemoresistant disease is common and can rarely be salvaged. *MYCN* gene amplification, found in 50% of high-risk patients, is associated with rapid tumour progression and a poor prognosis (reviewed by [10]). The overall long-term survival of high-risk patients currently remains less than 50%, with survivors often having long-term toxicities as a consequence of the intensive chemotherapy. Thus there is a continuing need to identify novel and less toxic therapies to improve survival of this subset of patients.

In neuroblastoma p53 mutations are rare, even at relapse (< 15%), and inactivation of the p53/MDM2/p14^ARF^ pathway in relapsed neuroblastoma is predominantly due to lesions upstream of p53, such as *MDM2* amplification and *p14^ARF^* aberrations [11]. Non-genotoxic activation of wt p53 using MDM2-p53 antagonists offers a novel therapeutic strategy for neuroblastoma treatment. Acquisition of resistance through *de novo* mutations following continuous exposure to Nutlin-3 have however been reported *in vitro*, and may limit the usefulness of MDM2-p53 antagonists as single-agent therapy [12]. This provides a rationale for using MDM2-p53 antagonists to improve the therapeutic index of current chemotherapy regimens, to enhance tumour killing without increasing toxicity whilst minimising the development of resistance. Studies to date have demonstrated the efficacy of Nutlin-3 in pre-clinical neuroblastoma models alone and in combination with cisplatin, camptothecin and bleomycin, and with targeted agents including bevacizumab and seliciclib (reviewed by [13]). However, the pre-clinical efficacy of the most advanced clinical candidate, RG7388, has yet to be evaluated in paediatric cancers, including neuroblastoma.

Using a panel of neuroblastoma cell lines, this study assessed the efficacy of RG7388 as a single agent, and in combination with chemotherapies routinely used to treat neuroblastoma, namely, cisplatin, doxorubicin, topotecan, temozolomide and busulfan. The overall aim was to provide pre-clinical data to support the clinical evaluation of RG7388 alone and/or in combination with conventional chemotherapy in patients with neuroblastoma to improve outcome and reduce toxicity.

## RESULTS

### RG7388 is highly potent in p53 wt neuroblastoma cell lines

**Figure 1:**
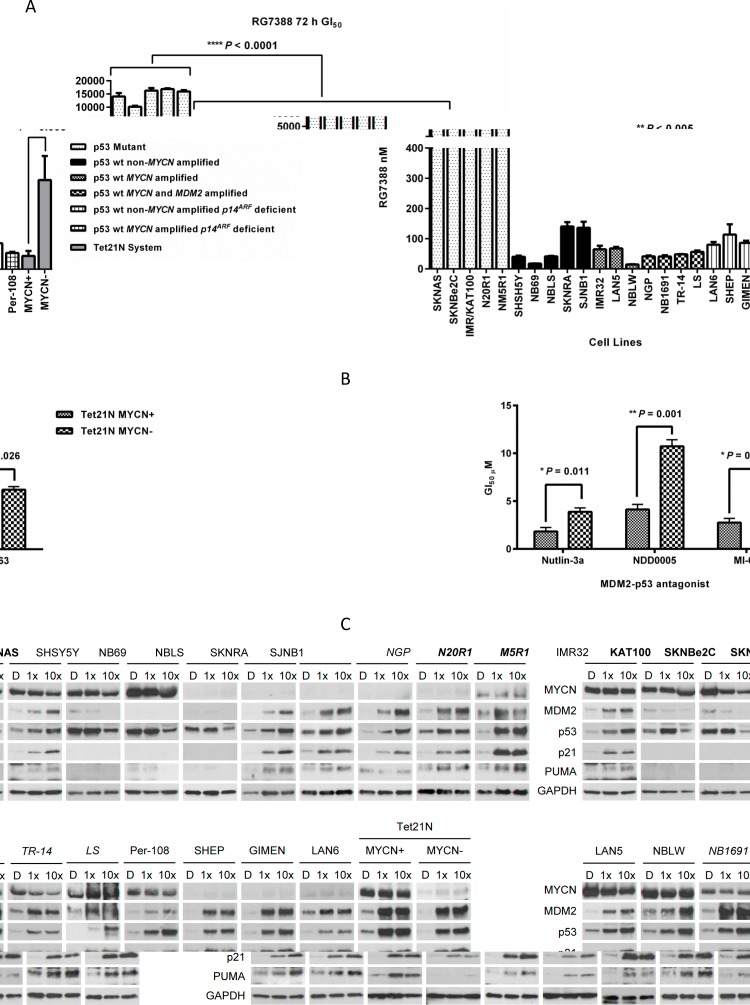
(A) Sensitivity of a panel of neuroblastoma cell lines of varying *MYCN*, MDM2, *p5*3 and *p14*^ARF^ status to RG7388 treatment for 72 hours. p53 wt cell lines are significantly more sensitive to RG7388 treatment *versus* p53 mutant cell lines (Mann Whitney test, *P* < 0.0001), and Tet21N MYCN+ cells are significantly more sensitive to RG7388 compared with Tet21N MYCN− cells (paired *t* test, *P* < 0.005). Data are shown as the average of at least 3 independent experiments and error bars represent SEM. (B) The sensitivity of Tet21N MYCN+ and MYCN− cells to MDM2 antagonists, Nutlin-3a, NDD0005 and MI-63. Tet21N MYCN+ cells are significantly more sensitive to Nutlin-3a (paired *t* test, *P* < 0.05), NDD0005 (paired *t* test, *P* < 0.005) and MI-63 (paired *t* test, *P* < 0.05) treatment for 72 hours compared with Tet21N MYCN− cells. Data shown are the average of at least 3 independent experiments and error bars represent SEM. (C) RG7388 treatment leads to stabilisation of p53 and upregulation of p53 targets, MDM2, p21 and PUMA in p53 wt but not p53 mutant neuroblastoma cell lines. Western analysis for activation of the p53 pathway in the panel of neuroblastoma cell lines and the p53 wt MYCN regulatable SHEP Tet21N cells in response to treatment for 6 hours with 1× and 10× their respective RG7388 GI_50_ concentrations. p53 mutant cell lines are represented in bold font and *MDM2* amplified cell lines are represented in italics. D, DMSO treated control cells.

The concentration of RG7388 required to inhibit growth by 50% (GI_50_) was determined using XTT cell proliferation assays in a panel of neuroblastoma cell lines, including 5 p53 mutant and 16 p53 wt cell lines of varying *MYCN*, *MDM2* and *p14^ARF^* status, together with the p53 wt MYCN− regulatable SHEP Tet21N cells (Table 1, Figure 1A, Supplementary Figure 1A). The panel included 2 isogenic paired p53 wt and mutant cell lines, IMR32 and IMR/KAT100, and NGP, N_N20R1 and N_M5R1. p53 wt, *MDM2* amplified human osteosarcoma SJSA-1 cells, previously shown to be sensitive to RG7388 and extensively used in the pre-clinical evaluation of several classes of MDM2-p53 antagonists to date, were used as a positive control [6, 8, 14-17] (Table 1). Consistent with the mechanism of action of MDM2-p53 antagonists, p53 wt neuroblastoma cell lines were significantly more sensitive to RG7388 compared to p53 mutant cell lines (*P* < 0.0001, Mann-Whitney test). Overall, all 16 neuroblastoma cell lines with wt p53 had nanomolar range GI_50_ values (range 14.8-140.3 nM; 68.2 (mean) ± 43.3 (SD) nM) of comparable sensitivity to SJSA-1 cells. In contrast, all 5 p53 mutant cell lines had GI_50_ values greater than 10 μM (range 10.1-16.9 μM; 14.6 (mean) ± 2.7 (SD) μM) (Table 1 and Figure 1A), representing > 200-fold differential between the average GI_50_ concentrations of p53 wt *versus* p53 mutant cell lines. Comparisons of GI_50_ concentrations between paired isogenic p53 wt and mutant neuroblastoma cell lines, demonstrated a 252-fold differential between IMR32 and IMR/KAT100, and a 406-fold and 384-fold differential between NGP and N_N20R1, and NGP and N_M5R1, respectively.

**Table 1:** GI_50_ concentrations for RG7388 in control osteosarcoma SJSA-1 cells and a panel of 21 neuroblastoma cell lines of varying *p53*, *MYCN*, *MDM2*, and *p14^ARF^* status, and the MYCN− regulatable Tet21N cells

Cell Line	*p53* Status	*MYCN* Status	*MDM2* Status	*p14*^ARF^ Deln/Meth	Cell Type	RG7388 (nM)
SJSA-1	Wt	Non-Amp	Amp		-	23.7 ± 1.5
SKNAS	Mut	Non-Amp	Non-Amp		S	10133 ± 240.8
SKNBe2C	Mut	Amp	Non-Amp		I	14040.8 ± 656.7
IMR/KAT100	Mut	Amp	Non-Amp		N	16291 ± 470.5
N_N20R1	Mut	Amp	Amp		N	16851.3 ± 161.9
N_M5R1	Mut	Amp	Amp		N	15926.7 ± 307.7
SHSY5Y	Wt	Non-Amp	Non-Amp		N	40.0 ± 3.9
NB69	Wt	Non-Amp	Non-Amp		N>S	17.5 ± 0.4
NBLS	Wt	Non-Amp	Non-Amp		S	41.6 ± 1.6
SKNRA	Wt	Non-Amp	Non-Amp		S	140.3 ± 14.7
SJNB1	Wt	Non-Amp	Non-Amp		S>N	136.2 ± 19.9
IMR32	Wt	Amp	Non-Amp		N	64.7 ± 6.0
LAN5	Wt	Amp	Non-Amp		N	68.0 ± 1.9
NBLW	Wt	Amp	Non-Amp		N>S	14.8 ± 0.3
NGP	Wt	Amp	Amp		N	41.5 ± 2.8
NB1691	Wt	Amp	Amp		N	41.0 ± 4.1
TR-14	Wt	Amp	Amp		N	48.2 ± 0.8
LS	Wt	Amp	Amp		N	56.3 ± 4.6
LAN6	Wt	Non-Amp	Non-Amp	Deleted	N	80.0 ± 9.1
SHEP	Wt	Non-Amp	Non-Amp	Deleted	S	113.8 ± 33.9
GIMEN	Wt	Non-Amp	Non-Amp	Methylated	S>N	86.1 ± 7.3
Per-108	Wt	Amp	Non-Amp	Methylated	N	53.7 ± 3.7
Tet21N MYCN−	Wt	Non-Amp	Non-Amp	Deleted	S	295.2 ± 39.7
Tet21N MYCN+	Wt	Non-Amp	Non-Amp	Deleted	S	44.1 ± 8.2

Data represents the mean of at least 3 independent experiments ± SEM. *p14*^ARF^ Deln/Meth, *p14*^ARF^ deleted/methylated; Wt, wild type; Mut, mutant; Non-Amp, non-amplified; Amp, amplified; N, neurite bearing (N-type); S, substrate adherent (S-type); I, intermediate (I-type)

### *MYCN, MDM2* and *p14*^ARF^ status and sensitivity to RG7388

*MYCN*, *p14^ARF^* and *MDM2* status have previously been linked to sensitivity to MDM2-p53 antagonists [18, 19]. In the isogenic Tet21N system, Tet21N MYCN+ cells were significantly more sensitive to RG7388 compared with Tet21N MYCN− cells (*P* < 0.005, paired *t* test, Figure 1A). Further studies found that Tet21N MYCN+ cells were also significantly more sensitive to other classes of MDM2-p53 antagonists, namely Nutlin-3a (*cis*-imidazoline) (*P* < 0.05, paired *t* test), NDD0005 (isoindolinone) (*P* < 0.005, paired *t* test) and MI-63 (spiro-oxindole) (*P* < 0.05, paired *t* test), compared with Tet21N MYCN− cells (Figure 1B). Sensitivity of the present panel of neuroblastoma cell lines to RG7388 was analysed in relation to their *MYCN, p14^ARF^* and *MDM2* status (Table 1 and Supplementary Figure 1B-E). The present panel included 8 *MYCN* amplified and 8 non-*MYCN* amplified p53 wt neuroblastoma cell lines (Table 1), and there was a non-significant trend for *MYCN* amplified cell lines to be more sensitive to RG7388 (*P* = 0.087, Welch *t* test, Supplementary Figure 1B).

Four out of 16 of the panel had *p14^ARF^* aberrations and 4/16 had non-syntenic amplification of *MDM2* and *MYCN* (Table 1 and Supplementary Figure 1A). Of note, *p14^ARF^* and *MDM2* abnormalities were mutually exclusive. There was a non-significant trend for *p14^ARF^* aberrant cell lines to be more resistant to RG7388 (*P* = 0.187, Welch *t* test, Supplementary Figure 1C). Similarly, there was a non-significant trend for *MDM2/MYCN* co-amplified cell lines to be more sensitive to RG7388 *versus* cell lines which were not *MDM2/MYCN* co-amplified (*P* = 0.074, Welch *t* test, Supplementary Figure 1D). No difference in sensitivity to RG7388 was found between *MDM2/MYCN* co-amplified *versus MYCN* amplified (*P* = 0.797, Welch *t* test, Supplementary Figure 1E).

### Functional activation of the p53 pathway in p53 wt neuroblastoma cell lines in response to RG7388 treatment

In the same panel of 21 neuroblastoma cell lines and the Tet21N system, functional activation of the p53 pathway in response to treatment for 6 hours with RG7388 at 1× and 10× their respective GI_50_ concentrations (Table 1) were assessed by Western blotting (Figure 1C). Stabilisation of p53, and induction of p53 targets, MDM2, p21^WAF1^ and PUMA were observed in all 16 p53 wt cell lines and Tet21N MYCN+ and MYCN− cells (Figure 1C), and in some cases this occurred in a concentration-dependent manner. Tet21N MYCN+ cells had higher basal p53 levels, as previously reported [20], and in response to RG7388 treatment exhibited higher levels of p53 stabilisation and had higher levels of MDM2 and PUMA compared with Tet21N MYCN− cells (Figure 1C). As expected, no induction of p53 or p53 targets was observed in p53 mutant neuroblastoma cell lines. All p53 mutant cell lines demonstrated a decrease in p53 expression in response to treatment with 10× their respective GI_50_ concentrations of RG7388, which is most likely attributable to the very high concentrations of RG7388 affecting cell viability due to off-target effects (Figure 1C).

### RG7388 induces cell cycle arrest and apoptosis in p53 wt neuroblastoma cell lines

**Figure 2:**
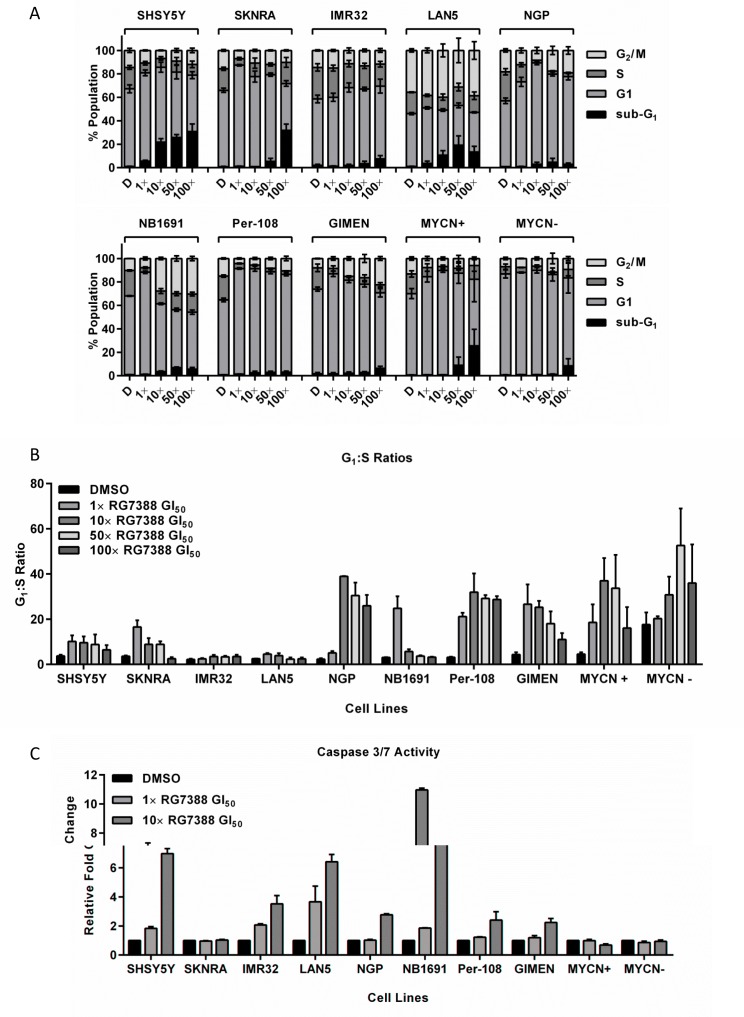
RG7388 treatment induces cell cycle arrest and/or apoptosis in p53 wt neuroblastoma cell lines Sub-G_1_ and cell cycle phase distribution (A) and G_1_:S ratios (B) of 8 *p53* wt neuroblastoma cell lines and the MYCN regulatable SHEP Tet21N cells treated for 24 hours with 1×, 10×, 50× or 100× their respective RG7388 GI_50_ concentrations. (C) Caspase 3/7 activity in the same panel of cell lines in response to 24 hours treatment with 1× or 10× their respective RG7388 GI_50_ concentrations or an equal volume of DMSO. Data are expressed as fold change relative to DMSO control, and are shown as the average of at least 3 independent experiments and error bars represent SEM. D, DMSO treated control cells; MYCN+, Tet21N MYCN+; MYCN−, Tet21N MYCN−.

From the original panel of cell lines assessed above, 8 p53 wt neuroblastoma cell lines (non-*MYCN* amplified SHSY5Y & SKNRA; *MYCN* amplified IMR32 & LAN5; *MDM2* and *MYCN* co-amplified NGP & NB1691; *p14^ARF^* methylated Per-108 & GIMEN) and Tet21N cells were analysed for cell cycle phase distribution and induction of apoptosis in response to RG7388 (Figure 2 and Supplementary Table 1). Cells were treated for 24 hours with 1×, 10×, 50× and 100× their respective GI_50_ concentrations of RG7388 (Table 1) and analysed using flow cytometry. An increase in the percentage of sub-G_1_ events, as a surrogate marker of apoptosis, was observed in all cell lines in response to one or more concentrations of RG7388. Overall, the accumulation of events in sub-G_1_ phase occurred in a concentration-dependent manner (Figure 2A and Supplementary Table 1). The G_1_:S ratio was calculated as an indicator of G_1_ cell cycle arrest, and with the exception of *MYCN* amplified LAN5 and IMR32 cells, all other cell lines including Tet21N cells in the presence and absence of MYCN, demonstrated at least a 2-fold increase in G_1_:S ratio in response to treatment with at least one or more concentrations of RG7388 (Figure 2B and Supplementary Table 1). In line with the role of MYCN in driving proliferation, switching off MYCN in the Tet21N system led to an increase in baseline G_1_:S ratio (MYCN+, 4.2 ± 0.8 *versus* MYCN−, 17.6 ± 5.3) (Figure 2B and Supplementary Table 1). Finally, treatment with ≥ 10× GI_50_ concentrations of RG7388 led to an accumulation of cells in G_2_/M phase in NGP, NB1691 and GIMEN cells (Figure 2A and Supplementary Table 1).

RG7388 induced apoptosis was also assessed by Caspase 3/7 assays in the 8 cell lines following treatment for 24 hours with 1× and 10× their respective RG7388 GI_50_ concentrations (Figure 2C). With the exception of SKNRA and Tet21N cells, all remaining p53 wt neuroblastoma cell lines exhibited a concentration-dependent increase in caspase 3/7 activity in response to RG7388 (Figure 2C). In Tet21N cells, MYCN+ cells had consistently higher caspase 3/7 activity compared with MYCN− cells (data not shown). In the majority of cell lines, increases in caspase 3/7 activity (Figure 2C) were consistent with the accumulation of sub-G_1_ events (Figure 2A and Supplementary Table 1), although caspase 3/7 activity is a more sensitive and specific indicator of apoptosis.

### RG7388 synergises with chemotherapies currently used to treat neuroblastoma

The current treatment of high-risk neuroblastoma involves combinations of different chemotherapies, including cisplatin, doxorubicin, topotecan, temozolomide and busulfan. Median-effect analysis was used to determine whether RG7388 can synergise with these chemotherapies in non-*MYCN* amplified SHSY5Y, *MYCN* amplified LAN5, and *MDM2* and *MYCN* co-amplified NGP, LS and NB1691 p53 wt neuroblastoma cell lines. With the exception of LAN5 cells which were established at diagnosis, all other cell lines were established post-treatment or at relapse. As part of median-effect analysis, the sensitivity of these p53 wt cell lines to 72 hours exposure to RG7388 and the above chemotherapies were determined. GI_50_ concentrations for RG7388 and the chemotherapies are shown in Tables 1 and 2, respectively. The sensitivity of the neuroblastoma cell lines in response to treatment with the tested chemotherapies alone demonstrated no obvious differences in GI_50_ concentrations across the cell lines to cisplatin or topotecan. LAN5 and NGP cells were more sensitive to doxorubicin compared to SHSY5Y, LS and NB1691 cells. Consistent with overexpression of methylguanine-DNA methyltransferase (MGMT), a known mechanism of temozolomide resistance, NGP cells which lack MGMT expression were the most sensitive to temozolomide (Supplementary Figure 1A). NB1691 cells were the most resistant to both temozolomide and busulfan.

**Figure 3:**
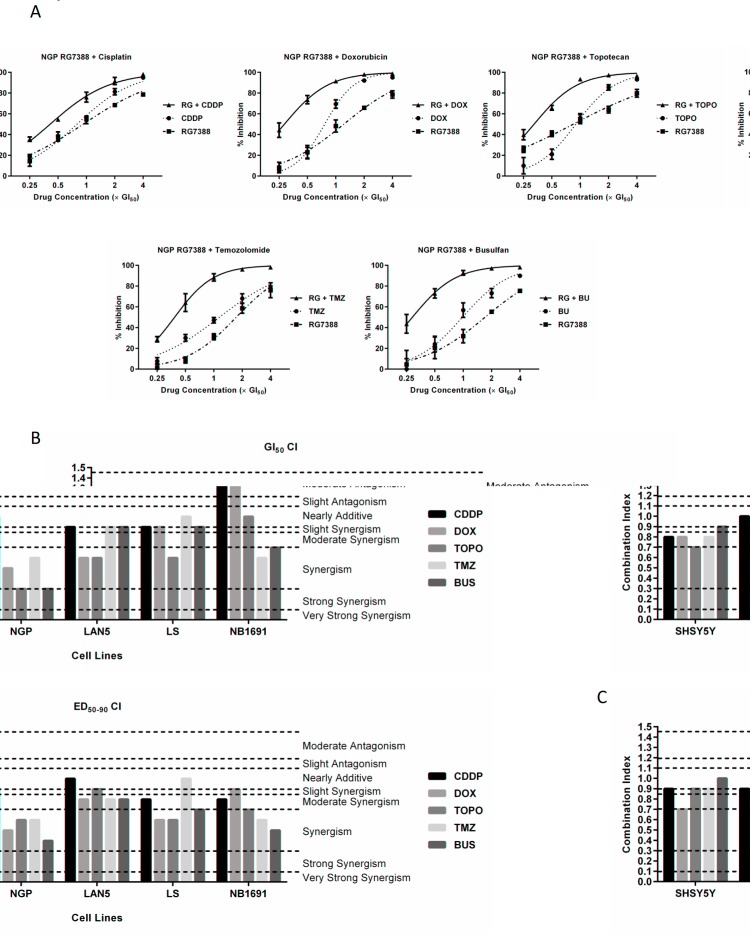
RG7388 synergises with cytotoxic chemotherapies in neuroblastoma cell lines (A) Growth inhibition curves of p53 wt NGP cells exposed to RG7388 and chemotherapy agents (cisplatin, doxorubicin, topotecan, temozolomide and busulfan) alone, and in combination at constant 1:1 ratios of 0.25×, 0.5×, 1×, 2× and 4× their respective GI_50_ concentrations for 72 hours. Data are shown as the average of at least 3 independent experiments and error bars represent SEM. (B) CI values of RG7388 in combination with chemotherapy agents at 1× their respective GI_50_ concentrations and (C) the average of CI values of RG7388 in combination with chemotherapy agents at effect levels ED_50_, ED_70_ and ED_90_ in p53 wt SHSY5Y, NGP, LAN5, LS and NB1691 neuroblastoma cell lines. RG, RG7388; Cisplatin, CDDP; doxorubicin, DOX; topotecan, TOPO; temozolomide, TMZ; busulfan, BU.

For combination studies, cells were treated for 72 hours with RG7388 and chemotherapies alone, and in combination at 5 equipotent concentrations between 0.125× and 4× their respective GI_50_ concentrations depending on the cell line and chemotherapy agent (Table 1 and 2). At least one or more of the *in vitro* RG7388 and chemotherapy concentrations tested were within previously observed clinically achievable peak plasma drug concentrations. Combination treatment in all cases led to greater growth inhibition compared to either treatment alone (Figure 3A and Supplementary Figures 2 & 3). The data were then analysed to determine whether the observed greater growth inhibition was additive or synergistic using median-effect analysis, which enables the quantitative evaluation of drug interactions based on the CI value. CI values were computed for each constant ratio combination and at estimated effect levels of ED_50_, ED_75_ and ED_90_ (Table 2 and Figure 3B & C). Overall, across all cell lines and chemotherapies, CI values varied with the effect level, therefore the average of CI values at ED_50_, ED_75_ and ED_90_ was also determined (Table 2 and Figure 3C). The majority of RG7388 and chemotherapy combinations at the GI_50_ concentrations ranged from slightly synergistic to synergistic (Figure 3B). The latter was also true when the average CI values at ED_50_, ED_75_ and ED_90_ was used to evaluate the interaction (Figure 3C). Taken together, cisplatin demonstrated the least degree of synergy with RG7388, whilst the remaining chemotherapies demonstrated comparable synergy with RG7388 (Table 2 and Figure 3B & C). With the exception of cisplatin, the most synergistic interactions between RG7388 and chemotherapy were observed in p53 wt, *MDM2* and *MYCN* co-amplified NGP cells (Table 2 and Figure 3). This observation did not however extend to two additional *MDM2* and *MYCN* co-amplified cell lines, LS and NB1691 (Table 2, Figure 3B & C and Supplementary Figure 3). RG7388 in combination with cisplatin and doxorubicin at their respective GI_50_ concentrations showed moderate antagonism in the NB1691 cells, but this was not observed when the average of CI values at ED_50_, ED_75_ and ED_90_ were taken into account (Table 2 and Figure 3B & C).

**Table 2:** GI_50_ concentrations for chemotherapy agents, and the CI values for RG7388 in combination with cisplatin, doxorubicin, topotecan, temozolomide or busulfan in *p53* wt SHSY5Y, NGP and LAN5 neuroblastoma cells GI_50_ concentrations are shown as the mean of at least 3 independent experiments ± SEM. RG7388 was combined with chemotherapy at the indicated fixed 1:1 ratios relative to their respective GI_50_ concentrations. CI values were calculated for each constant ratio combination and at effect levels ED_50_, ED_75_ and ED_90_ from the average of at least 3 independent experiments. CI Ave ED_50-90_ represents the average of CI values at effect levels ED_50_, ED_75_ and ED_90_. CI range: < 0.1 very strong synergism; 0.1-0.3 strong synergism; 0.3-0.7 synergism; 0.7-0.85 moderate synergism; 0.85-0.9 slight synergism; 0.9-1.1 nearly additive; 1.1-1.2 slight antagonism; 1.2-1.45 moderate antagonism; 1.45-3.3 antagonism; 3.3-10 strong antagonism; > 10 very strong antagonism.

Cell Line	Chemotherapy	GI_50_	CI						CI ED_50_	CI ED_75_	CI ED_90_	CI Ave ED_50-90_
			× GI_50_									
			0.125	0.25	0.5	1	2	4				
SHSY5Y^1^	Cisplatin	0.7 ± 0.1 μM	ND	0.8	0.8	**0.8**	1	1	0.8	0.9	0.9	**0.9**
	Doxorubicin	30.7 ± 2.7 nM	ND	0.9	0.8	**0.8**	0.6	0.4	0.8	0.7	0.6	**0.7**
	Topotecan	9.5 ± 0.2 nM	ND	1.1	0.9	**0.7**	0.8	1.2	0.9	0.9	0.9	**0.9**
	Temozolomide	331.5 ± 10.2 μM	ND	0.5	0.6	**0.8**	1.1	1.7	0.7	0.9	1.2	**0.9**
	Busulfan	26.21 ± 4.8 μM	ND	0.7	0.7	**0.9**	1.3	1.3	0.8	1	1.3	**1**
NGP^2^	Cisplatin	1.1 ± 0.1 μM	ND	0.9	1	**1**	0.8	0.5	1	0.9	0.7	**0.9**
	Doxorubicin	9.6 ± 0.9 nM	ND	0.6	0.6	**0.5**	0.5	0.8	0.5	0.5	0.6	**0.5**
	Topotecan	9.9 ± 0.3 nM	ND	0.8	0.7	**0.3**	0.4	0.8	0.6	0.5	0.5	**0.6**
	Temozolomide	16.4 ± 3.7 μM	ND	0.4	0.5	**0.6**	0.8	1.5	0.5	0.6	0.8	**0.6**
	Busulfan	59.3 ± 3.1 μM	ND	0.5	0.4	**0.3**	0.3	0.4	0.4	0.4	0.4	**0.4**
LAN5^1^	Cisplatin	0.5 ± 0.1 μM	ND	0.9	0.9	**0.9**	1	1.5	0.9	1	1.1	**1**
	Doxorubicin	11.7 ± 0.6 nM	ND	0.8	0.7	**0.6**	0.9	1.8	0.7	0.8	1	**0.8**
	Topotecan	8.1 ± 0.7 nM	ND	1.1	1	**0.6**	0.8	1.3	0.9	0.9	1	**0.9**
	Temozolomide	174.3 ± 13.3 μM	ND	0.4	0.6	**0.9**	1.2	2	0.6	0.8	1	**0.8**
	Busulfan	66.0 ± 2.5 μM	ND	0.6	0.8	**0.9**	1	0.9	0.8	0.8	0.9	**0.8**
LS^1^	Cisplatin	1.2 ± 0.3 μM	ND	0.8	1	**0.9**	0.8	0.8	0.8	0.8	0.8	**0.8**
	Doxorubicin	42.4 ± 5.9 nM	ND	0.8	0.7	**0.9**	0.9	< 0.1	1	0.5	0.3	**0.6**
	Topotecan	9.0 ± 0.3 nM	ND	0.6	0.5	**0.6**	0.8	0.6	0.6	0.6	0.6	**0.6**
	Temozolomide	525.6 ± 21.5 μM	1.6	1	0.7	**1**	1.2	ND	1.1	1	1	**1**
	Busulfan	185.9 ± 8.8 μM	ND	0.5	0.7	**0.9**	1	0.6	0.6	0.7	0.8	**0.7**
NB1691^1^	Cisplatin	2.3 ± 0.1 μM	ND	1.1	1.3	**1.3**	0.7	0.2	1.1	0.8	0.6	**0.8**
	Doxorubicin	23.4 ± 2.6 nM	ND	1	1.1	**1.3**	0.9	0.3	1.1	0.8	0.7	**0.9**
	Topotecan	8.1 ± 0.2 nM	ND	0.7	0.8	**1**	0.9	0.2	0.9	0.7	0.6	**0.7**
	Temozolomide	868.1 ± 10.8 μM	1.1	1	0.9	**0.6**	0.4	ND	0.8	0.6	0.5	**0.6**
	Busulfan	693.4 ± 38.6 μM	0.7	1	1	**0.7**	< 0.1	ND	0.9	0.4	0.2	**0.5**

1 high MGMT expression;

2 low MGMT expression (see supplementary Figure 1A); ND, not determined

### RG7388 in combination with chemotherapies leads to increased apoptosis

**Figure 4:**
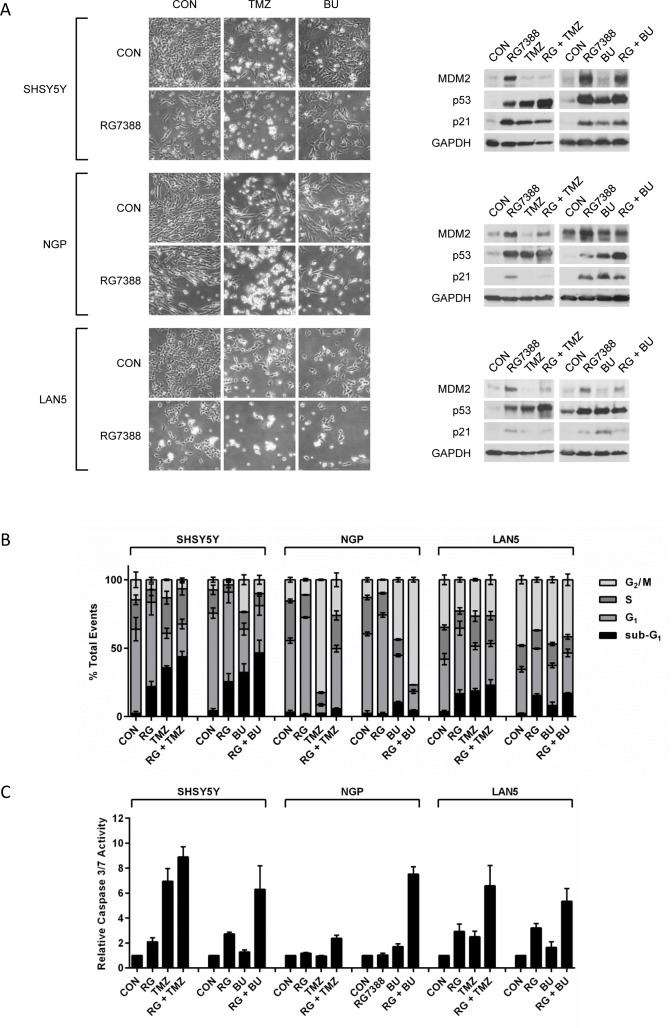
Combinations of RG7388 with temozolomide or busulfan leads to increased apoptosis in p53 wt neuroblastoma cells p53 wt SHSY5Y, NGP and LAN5 cells were treated with their respective GI_50_ concentrations of RG7388 and chemotherapy agent alone, and in combination, and assessed at 72 hours post-treatment by (A) light-microscopy for morphological appearance and Western analysis for functional p53 pathway activation and (B) flow cytometry for sub-G_1_ and cell cycle phase distribution, and at 24 hours post-treatment for (C) caspase 3/7 activity as an indicator of apoptosis. Caspase 3/7 activity is represented as fold change relative to solvent control. CON, solvent control; RG, RG7388; TMZ, Temozolomide; BU, busulfan. Data are shown as the average of at least 3 independent experiments and error bars represent SEM.

Functional evaluation of RG7388 in combination with cisplatin, doxorubicin, topotecan, temozolomide and busulfan was performed in p53 wt SHSY5Y, NGP and LAN5 cells. Cells were treated with their respective GI_50_ concentrations for RG7388 and the chemotherapy agent alone, and in combination, and then assessed by light microscopy for morphological appearance, Western analysis for p53 pathway activation and flow cytometry for sub-G_1_ and cell cycle phase distribution at 72 hours post-treatment (Figure 4A & B and Supplementary Figure 4A & B). In addition, cells were also assessed for caspase 3/7 activity as an indicator of apoptosis at 24 hours post-treatment (Figure 4C and Supplementary Figure 4C). Overall, for all combination treatments, there was an increase in the number of detached cells compared to vehicle control and either treatment alone (Figure 4A and Supplementary Figure 4A). Western analysis demonstrated that treatment of p53 wt neuroblastoma cells with RG7388 and chemotherapy agents alone and in combination led to p53 stabilisation and activation of the p53 pathway (Figure 4A and Supplementary Figure 4A). Furthermore, combination treatment in almost all cases induced greater levels of p53 stabilisation compared to the chemotherapy agent alone, and in some cases this was also greater than those induced by RG7388 alone. In response to treatment with GI_50_ concentrations of RG7388 for 72 hours, SHSY5Y and LAN5 cells demonstrated an increase in both sub-G_1_ events and G_1_:S ratios, indicative of induction of apoptosis and a G_1_ cell cycle arrest, respectively (Figure 4B and Supplementary Figure 4B). In contrast, NGP cells only exhibited an increase in G_1_:S ratio (Figure 4B and Supplementary Figure 4B). Overall, at GI_50_ concentrations, RG7388 was more potent than the tested chemotherapy agents at consistently inducing a cell cycle arrest in SHSY5Y, NGP and LAN5 cells. Assessment of caspase 3/7 activity as a marker of apoptosis demonstrated that GI_50_ concentrations of RG7388 or chemotherapy agent alone led to an increase in activity levels in SHSY5Y and LAN5 cells (Figure 4C and Supplementary Figure 4C), however, only GI_50_ concentrations of busulfan and topotecan led to an increase in activity levels in NGP cells. Overall, combination treatments led to greater caspase 3/7 activity in p53 wt neuroblastoma cells compared to vehicle control and single treatment alone, suggesting enhanced tumour cell killing (Figure 4C and Supplementary Figure 4C). In addition, in the majority of cases, increases in caspase 3/7 activity at 24 hours post-treatment (Figure 4C and Supplementary Figure 4C) were consistent with the accumulation of sub-G_1_ events 72 hours post-treatment (Figure 4B and Supplementary Figure 4B).

## DISCUSSION

Chemotherapy remains an essential component of multimodal cancer treatment, but can result in severe short and long-term toxicity. In the modern era of cancer therapeutics there is a drive towards the identification and use of targeted agents to enhance cancer specific killing while reducing toxicity. The use of MDM2-p53 antagonists represents a potential novel therapeutic strategy in neuroblastoma, where the incidence of p53 mutations is much lower in comparison to adult malignancies and p53 mutations are rare even at relapse (reviewed by [13]). This study demonstrates for the first time the highly selective and potent *in vitro* anti-tumour activity of RG7388 as a single agent in p53 wt neuroblastoma of varying *MYCN*, *MDM2* and *p14^ARF^* genetic status, resulting in p53 stabilisation and activation of the p53 pathway.

Within the present panel of p53 wt neuroblastoma cell lines, the highest RG7388 GI_50_ concentrations (≥ 80 nM) were obtained in *MYCN* non-amplified cell lines of which 4/5 cell lines (SKNRA, SJNB1, SHEP and GIMEN) comprise predominantly S-type (substrate adherent) cells [21] and 3 cell lines (SHEP, GIMEN and LAN6) have *p14^ARF^* aberrations [22]. These observations are consistent with previous studies showing that MYCN sensitises neuroblastoma cell lines to MDM2-p53 antagonists, Nutlin-3 and MI-63 [18], and p14^ARF^ silencing leads to resistance to Nutlin-3 induced apoptosis [19]. Furthermore, the reduced sensitivity of S-type cells to RG7388 compared to N-type (neuronal) cells is consistent with previous reports of different outcomes of S- *versus* N-type neuroblastoma cells in response to p53 activation, including Nutlin-3 and MI-63, where N-type cells are more likely to undergo apoptosis in contrast to S-type cells which undergo a G_1_ cell cycle arrest [18, 21, 23, 24]. In response to 10× GI_50_ concentrations of RG7388, the one predominantly S-type (S>N) and all the N-type neuroblastoma cell lines tested in the present study demonstrated increased caspase 3/7 activity, whereas S-type SKNRA cells did not.

The difference between the mean RG7388 GI_50_ concentrations of *MYCN* amplified *versus* non-amplified cell lines was not statistically significant, in contrast to our previous study with Nutlin-3 and MI-63 [18] but consistent with another [19]. However, in line with Gamble *et al* [18], sensitivity of *MYCN* non-amplified neuroblastoma cell lines to RG7388 were more varied compared with *MYCN* amplified cell lines. In the isogenic system, however, Tet21N MYCN+ cells were significantly more sensitive to RG7388, as well as to other structurally diverse MDM2-p53 antagonists, namely, NDD0005, MI-63 and Nutlin-3a, compared with Tet21N MYCN− cells. This result is consistent with previous studies of Nutlin-3 and MI-63 in this paired cell line [18, 25], and the greater sensitivity of Tet21N MYCN+ cells to chemotherapy [26]. Increased sensitivity of Tet21N cells to RG7388 and other MDM2-p53 antagonists in the presence of MYCN, may be explained by our previous observations that p53 is a direct transcriptional target of MYCN [20]. Consistent with this, the present study demonstrated higher basal p53 levels in Tet21N MYCN+ cells, as previously reported [20]. Furthermore, in response to RG7388 treatment there was greater p53 stabilisation and induction of PUMA compared with Tet21N MYCN− cells, despite being treated with a lower concentration of RG7388. *MDM2* has also previously been reported as a direct target gene of MYCN [27], however we did not observe any difference in RG7388 sensitivity between *MYCN* and *MDM2* co-amplified *versus MYCN* amplified cell lines in the present study, in contrast to Gamble *et al* [18].

RG7388 induced cell cycle arrest and/or apoptosis in all p53 wt neuroblastoma cell lines tested, and in most cases induction of apoptosis demonstrated by an increase in caspase 3/7 activity and sub-G_1_ events in a concentration-dependent manner. Cell cycle arrest was not always accompanied by induction of apoptosis, and overall, a higher concentration of RG7388 was required to induce apoptosis compared to a G_1_ arrest, consistent with previous observations and the p53 apoptotic threshold in p53 wt tumour cells [17, 28]. *MYCN* amplified cell lines, LAN5 and IMR32, were the only cell lines which did not demonstrate a ≥ 2-fold increase in G_1_:S ratios, indicative of a G_1_ cell cycle arrest, in response to treatment with at least one or more concentrations of RG7388. This latter result is in part consistent with our previous observations of a failure of *MYCN* amplified cell lines to undergo G_1_ arrest following irradiation-induced DNA damage [29]; however, this did not extend to the *MDM2* and *MYCN* co-amplified cell lines NGP and NB1691 as a ≥ 2-fold increase in G_1_:S ratio was observed.

Despite evidence of single-agent activity, the development of resistance mechanisms may ultimately limit the efficacy of MDM2-p53 antagonists given alone [12]. Most likely, MDM2-p53 antagonists will be used in combination, initially with existing chemotherapies and/or radiotherapy, and then with other novel targeted agents. The ideal combinations should lead to synergistic cancer cell killing while reducing the toxicity associated with intensive chemotherapy regimens presently used, which is particularly important in young children. To this end, the present study assessed whether RG7388 synergises with 5 chemotherapy agents most commonly used during frontline treatment at diagnosis or relapse, namely cisplatin, doxorubicin, topotecan (induction), busulfan (consolidation) and temozolomide (relapse) in 5 p53 wt neuroblastoma cell lines.

In particular, cisplatin is associated with nephrotoxicity and ototoxcitiy, topotecan and doxorubicin are associated with significant myelosuppression and high-dose doxorubicin with cardiotoxicity. Temozolomide is used in a refractory/relapsed disease setting, and high-dose busulfan is presently given with high-dose melphalan followed by autologous haematopoietic stem cell rescue in the consolidation phase of high-risk neuroblastoma treatment, and is associated with significant non-haematological side-effects including liver (veno-occlusive disease) and lung toxicity [30]. Overall, the majority of RG7388 and chemotherapy combinations ranged from slightly synergistic to synergistic in line with CI values observed by previous studies of Nutlin-3 and chemotherapy, in other cancer types [31, 32]. In most cases, combination treatment led to greater stabilisation of p53 and increased apoptosis, as evident by higher levels of caspase-3/7 activity compared to either agent alone. Furthermore, analysis of CI values against caspase 3/7 activity of combination treatments demonstrated a significant correlation between increased synergy and increased apoptosis (Spearman's Correlation, *P* < 0.0001) in SHSY5Y and NGP cells (Supplementary Figure 5).

Taking into consideration the toxicity profiles of the chemotherapeutics tested, and the reported cytopenias associated with RG7112/RG7388, the most rational and clinically relevant combinations of RG7388 are with temozolomide or busulfan. We propose that following Phase I evaluation of RG7388 alone, it is tested alongside a temozolomide backbone in a randomised Phase II setting possibly as an additional arm of the BEACON trial (www.clinicaltrials.gov; NCT01114555). In addition, RG7388 could be combined with lower than currently used doses of busulfan to try and reduce the non-haematological dose-limiting toxicities of busulfan, such as veno-occlusive disease of the liver and lungs [30]. Here myelosuppression is not a concern as busulfan is given immediately prior to autologous haematopoietic stem cell rescue.

Future *in vivo* studies to support observations of the present *in vitro* study, including dosing and scheduling, will need to be conducted in appropriate *in vivo* models of both efficacy and toxicity prior to clinical evaluations of RG7388 in neuroblastoma patients. For MDM2-p53 antagonists murine transgenic models may not be suitable as despite a high degree of homology between human and mouse MDM2 [33], MDM2-p53 antagonists display interspecies selectivity, with reduced binding affinities for mouse and rat MDM2 [34, 35]. Consistent with this, we have observed that cell lines derived from *MYCN* transgenic mice are less sensitive to MDM2-p53 antagonists than human neuroblastoma cell lines (*Chen et al, manuscript in preparation*).

In conclusion, the current study is the first to report the highly potent anti-tumour *in vitro* activity of RG7388 in p53 wt neuroblastoma cells as a single agent, and synergistic activity with conventional chemotherapies routinely used to treat neuroblastoma patients. This data supports the clinical evaluation of RG7388 alone or in combination, in particular with temozolomide or busulfan, as a novel therapeutic strategy to potentially improve survival and/or reduce toxicity of patients with neuroblastoma. The presence of wt p53 remains the most robust predictive biomarker of response to RG7388, however *MYCN*, *MDM2* and *p14^ARF^* status should also be recorded. Further identification and validation of non-invasive, reliable pharmacodynamic proof-of-mechanism tumour biomarkers of response to RG7388 are also necessary to support the clinical evaluation of this class of novel inhibitors in children with neuroblastoma.

## MATERIALS AND METHODS

### Chemicals

RG7388 was provided by Hoffman-La Roche (Nutley, NJ, USA) [6]. Nutlin-3a was purchased from Cambridge Bioscience Ltd (Cambridge, UK). MI-63 and NDD0005 were synthesised as previously described [36, 37]. Cisplatin (Merck Millipore, Watford, UK) was dissolved in dimethylformamide (DMF). Doxorubicin, topotecan, temozolomide and busulfan (Sigma-Aldrich, Dorset, UK) were dissolved in dimethyl sulfoxide (DMSO).

### Cell lines

Human neuroblastoma cell lines used and their *MYCN, p53, MDM2* and *p14^ARF^* genetic status are listed in Table 1. N_N20R1 and N_M5R1 were generated from parental NGP cells with resistance to 20 μM Nutlin-3 or 5 μM MI-63, respectively. Both cell lines harbour *p53* point mutations at codon 152 (exon 5) with N_M5R1 having a second point mutation at codon 176 (exon 5), and N_N20R1 having a second mutation at codon 98 (exon 4) (J. Lunec, unpublished). All neuroblastoma cell lines were obtained between 1996 and 2007 and were validated upon receipt using cytogenetic analysis courtesy of Dr Nick Bown (Institute of Human Genetics, Newcastle University), and maintained as previously described [38]. To switch off MYCN, Tet21N cells were cultured in the presence of 1 μg/mL of tetracycline (Sigma) for at least 24 hours. p53 wt, *MDM2* amplified human osteosarcoma SJSA-1 cells obtained from the ATCC were cultured in RPMI-1640 supplemented with 10% (v/v) FCS. Photomicrographs were captured using a VisiCam® digital camera and analyser software (VWR International Ltd, Lutterworth, UK).

### Growth inhibition assays and median-effect analysis

Seventy-two hour growth inhibition assays and GI_50_ concentration determination were performed as previously described [38]. For combination studies, cells were treated for 72 hours with RG7388 and conventional chemotherapies alone and in combination simultaneously at constant 1:1 ratios of 0.25×, 0.5×, 1×, 2× and 4×, or 0.125×, 0.25×, 0.5×, 1× and 2×, their respective GI_50_ concentrations, depending on the drug solubility. Median-effect analysis and Combination Index (CI) values were determined using CalcuSyn v2 (Biosoft, Cambridge, UK). Experiments were at least n=3.

### Western blotting

Western analysis was carried out as previously described [38]. Primary antibodies used were p53 1:1000 (NCL-L-p53-DO7, Leica Biosystems Ltd, Newcastle upon Tyne, UK), MYCN 1:500 (sc-53993, Santa Cruz Biotechnology Inc., Dallas, TX, USA), MDM2 1:200 (OP46, Merck), p21^WAF1^ 1:200 (OP64, Merck), p53 upregulated modulator of apoptosis (PUMA) 1:500 (ab9643, Abcam, Cambridge, UK) and GAPDH 1:500 (sc-25778, Santa Cruz). Experiments were at least n=3.

### Flow cytometry

Cells were harvested at the indicated times post-treatment, fixed in ice-cold 70% (v/v) ethanol and stored at −20°C. Prior to analysis, cells were washed with PBS, resuspended in 500 μL PBS with 50 μg/mL propidium iodide (Sigma) and 50 μg/mL RNAse A (Sigma), and incubated at 37°C for 30 minutes. Samples were analysed on the FACSCalibur^TM^ using CellQuest Pro software (Becton Dickinson, Oxford, UK). Data were analysed using Cyflogic (CyFlo Ltd, Turku, Finland). Experiments were at least n=3.

### Caspase 3/7 assays

Caspase-3/7 activity was assayed using Caspase-Glo 3/7 assay (Promega, Southampton, UK) according to the manufacturer's instructions. Experiments were at least n=3.

### Statistical analyses

All statistical tests were performed using GraphPad Prism v6.0 software and *P* < 0.05 taken to be the level of statistical significance.

## SUPPLEMENTARY MATERIAL, FIGURES AND TABLE


