# Supplementary material for: Pre-clinical evaluation of the MDM2-p53 antagonist RG7388 alone and in combination with chemotherapy in neuroblastoma
Source: Oncotarget. 2015 Mar 10;6(12):10207–21. doi: 10.18632/oncotarget.3504 (PMC4496350; doi:10.18632/oncotarget.3504)
Supplement: Supplementary file 1 [file oncotarget-06-10207-s001.pdf]

# Pre-clinical evaluation of the MDM2-p53 antagonist RG7388 alone and in combination with chemotherapy in neuroblastoma

## Supplementary Material

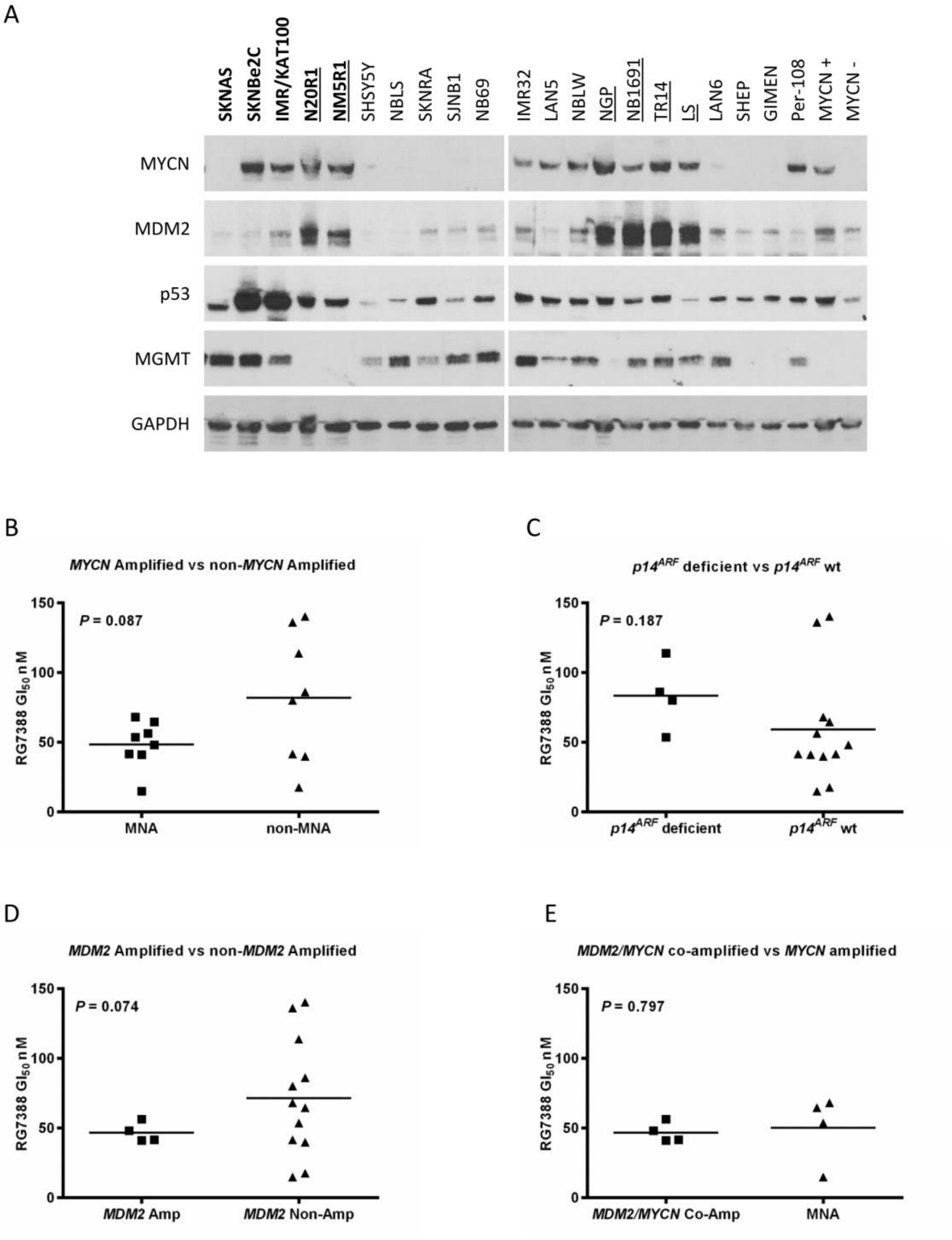


**Supplementary Figure 1: (A) Western analysis for basal expression levels of MYCN, MDM2, p53 and MGMT in the panel of 21 *p53* wt and mutant neuroblastoma cell lines of varying *MYCN*, *MDM2*, *p53* and *p14*$^{ARF}$ status, and MYCN regulatable Tet21N cells in the presence and absence of MYCN.** *p53* mutant cell lines are in bold font, and *MDM2* and *MYCN* co-amplified cell lines are

underlined. High MGMT expression is associated with resistance to temozolomide. Primary mouse anti-MGMT antibody (Chemicon International) was used at 1:1000 overnight at 4°C. **(B-E) The relationship between sensitivity to RG7388 and *MYCN*, *p14$^{ARF}$* and *MDM2* status in *p53* wt neuroblastoma cell lines.** Sensitivity to RG7388 in (B) *MYCN* amplified (MNA) *versus* non-*MYCN* amplified (non-MNA) cells; (C) *p14$^{ARF}$* deficient *versus* *p14$^{ARF}$* wt cells; (D) *MDM2* amplified *versus* non-*MDM2* amplified cells and; (E) *MDM2* and *MYCN* co-amplified *versus* *MYCN* amplified cells. Each data point represents the average of least 3 independent experiments and horizontal lines represent the mean. Two tailed, Welch unpaired *t* tests were used and statistical significance was taken as $P < 0.05$.

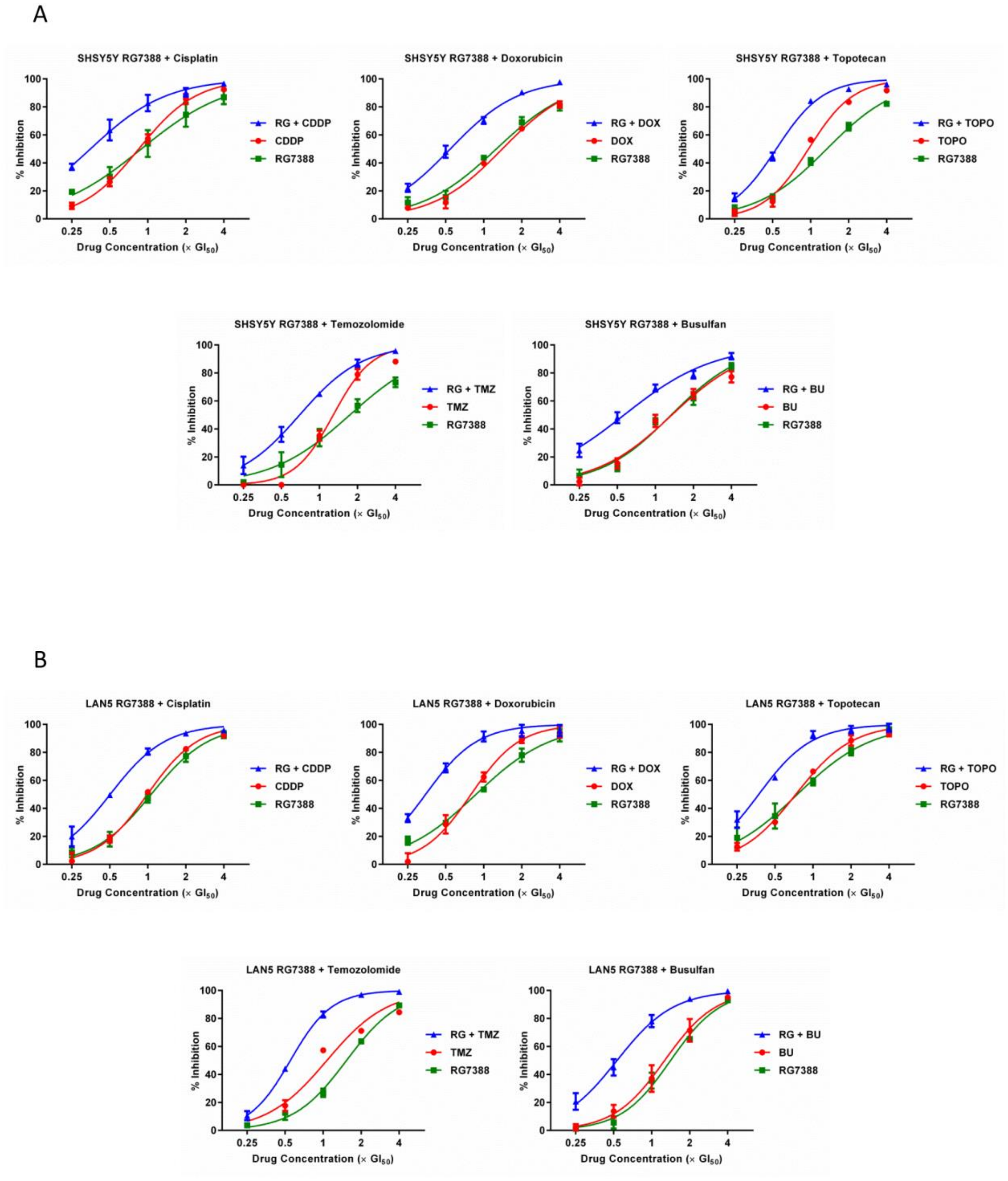


**Supplementary Figure 2: Sensitivity of *p53* wt SHSY5Y and LAN5 neuroblastoma cells to RG7388 and chemotherapy agents alone, and in combination.** 72 hour growth inhibition curves of (A) non-*MYCN* amplified SHSY5Y and (B) *MYCN* amplified LAN5 cells exposed to RG7388 and chemotherapy agents (cisplatin, doxorubicin, topotecan, temozolomide and busulfan) alone, and in combination at

the indicated constant 1:1 ratios relative to their respective $GI_{50}$ concentrations. RG, RG7388; Cisplatin, CDDP; doxorubicin, DOX; topotecan, TOPO; temozolomide, TMZ; busulfan, BU. Data are shown as the average of at least 3 independent experiments and error bars represent SEM.

A

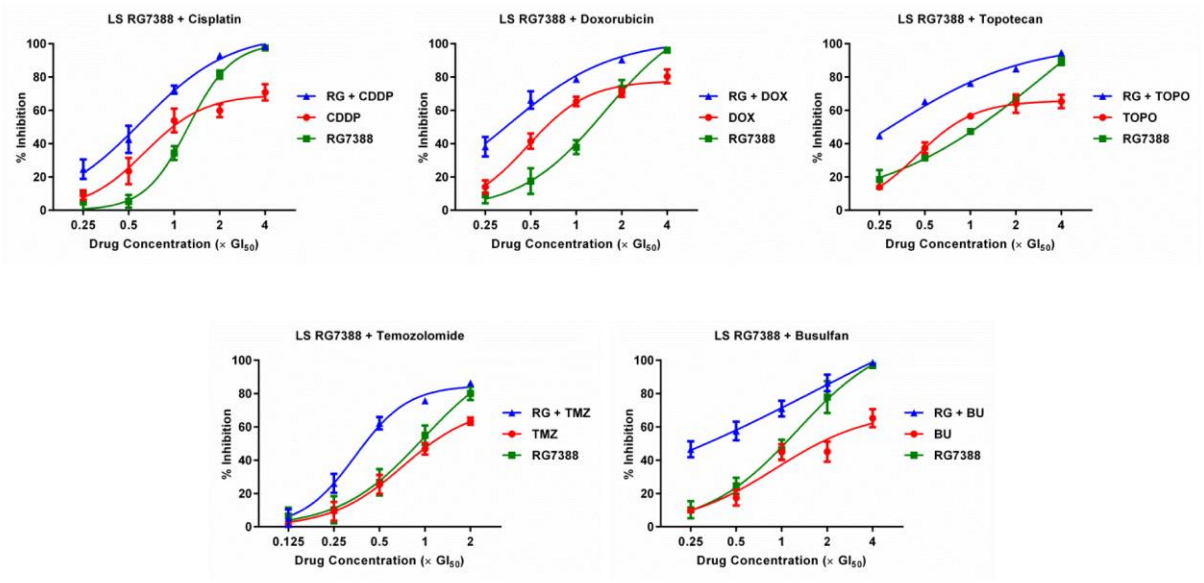


B

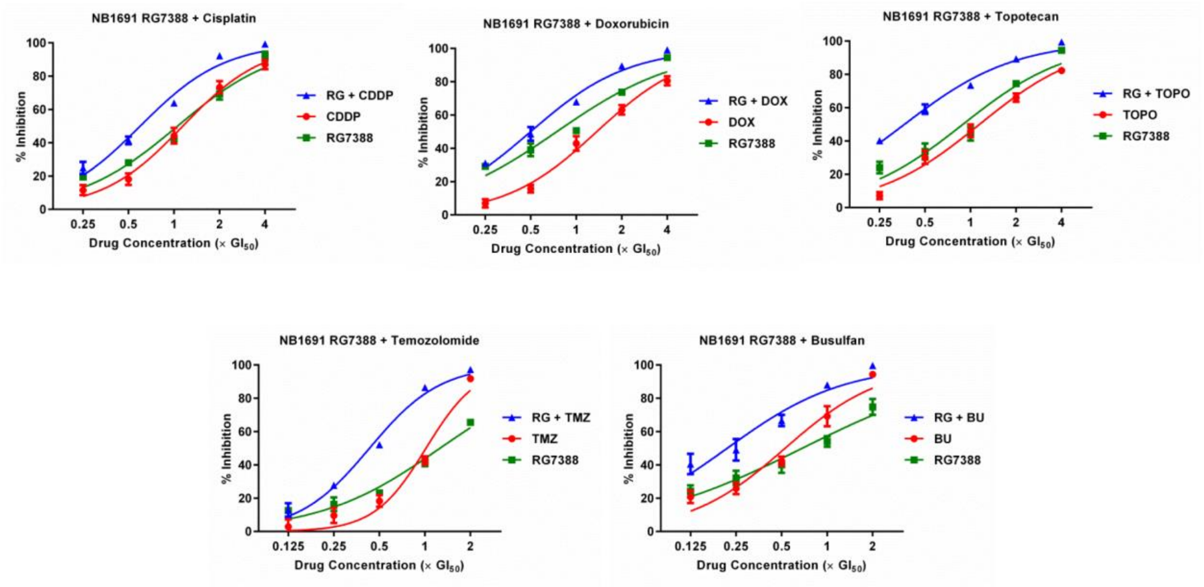


**Supplementary Figure 3: Sensitivity of *p53* wt, *MYCN and MDM2* co-amplified LS and NB1691 neuroblastoma cells to RG7388 and chemotherapy agents alone, and in combination.** 72 hour growth inhibition curves of (A) LS and (B) NB1691 cells exposed to RG7388 and chemotherapy agents

(cisplatin, doxorubicin, topotecan, temozolomide and busulfan) alone, and in combination at the indicated constant 1:1 ratios relative to their respective $GI_{50}$ concentrations. RG, RG7388; Cisplatin, CDDP; doxorubicin, DOX; topotecan, TOPO; temozolomide, TMZ; busulfan, BU. Data are shown as the average of at least 3 independent experiments and error bars represent SEM.

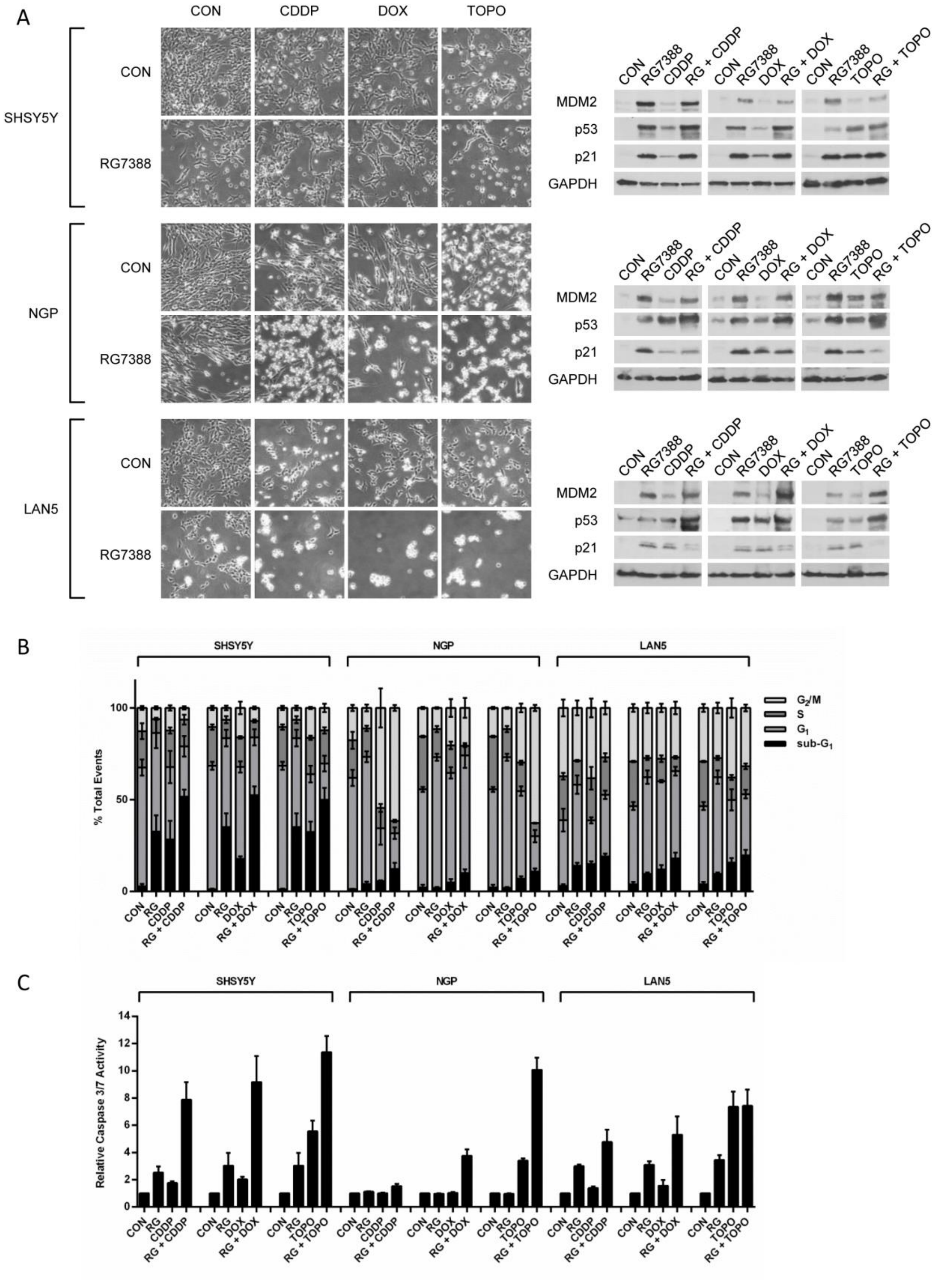


**Supplementary Figure 4: RG7388 in combination with cisplatin, doxorubicin and topotecan leads to increased apoptosis in *p53* wt neuroblastoma cells.** p53 wt SHSY5Y, NGP and LAN5 cells were treated with their respective $GI_{50}$ concentrations of RG7388 and chemotherapy agent alone, and in

combination, and assessed at 72 hours post-treatment by (A) light-microscopy for morphological appearance and Western analysis for functional p53 pathway activation and (B) flow cytometry for sub-$G_1$ and cell cycle distribution, and at 24 hours post-treatment for (C) caspase 3/7 activity as an indicator of apoptosis. Caspase 3/7 activity is represented as fold change relative to solvent control. CON, solvent control; RG, RG7388; CDDP, Cisplatin; DOX, Doxorubicin; TOPO, Topotecan. Data are shown as the average of at least 3 independent experiments and error bars represent SEM.

A

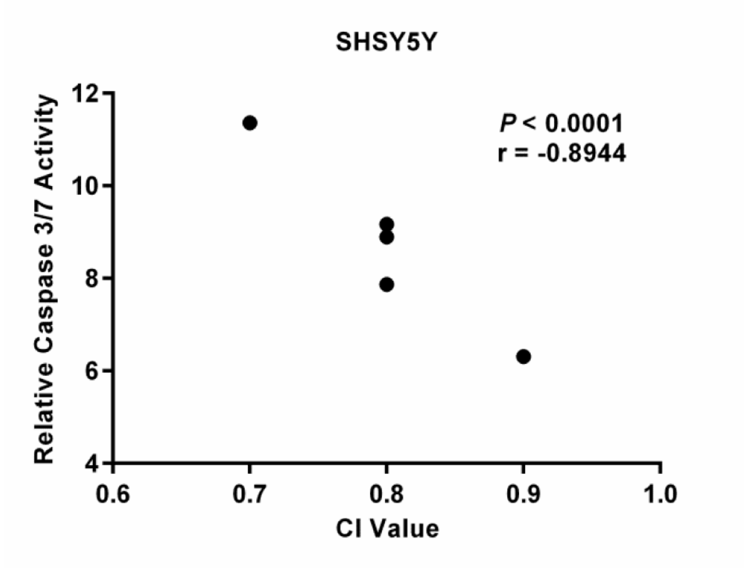


B

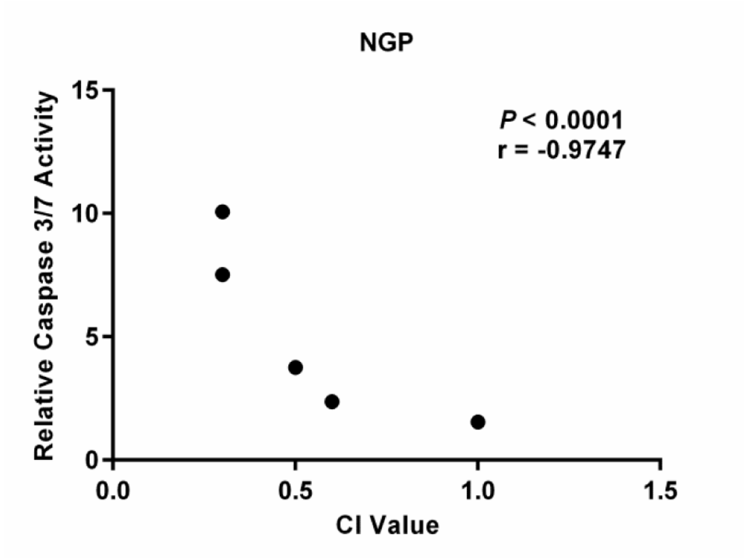


**Supplementary Figure 5: Correlation between degree of synergy and apoptosis in p53 wt SHSY5Y and NGP neuroblastoma cells.** Analysis of CI values versus relative caspase 3/7 activity of RG7388 and chemotherapy combination treatments at 1× $GI_{50}$ concentrations using Spearman's Correlation in (A) SHSY5Y and (B) NGP cells.

**Supplementary Table 1: Cell cycle distribution and $G_1$:S ratios of 8 selected p53 wt neuroblastoma cell lines and the Tet21N cells in the presence and absence of MYCN, treated with DMSO or 1×, 10×, 50× or 100× their respective RG7388 $GI_{50}$ concentrations for 24 hours.** Data represents the mean of at least 3 independent experiments ± SEM.

| Cell Line | Treatment | % Total Events | | | | $G_1$:S Ratio |
|---|---|---|---|---|---|---|
| | | sub-$G_1$ | $G_1$ | S | $G_2$/M | |
| SHSY5Y | DMSO | 1.1 ± 0.2 | 66.2 ± 3.4 | 18.2 ± 1.7 | 14.5 ± 1.7 | 3.7 ± 0.6 |
| | 1× | 5.6 ± 0.7 | 75.3 ± 2.5 | 8.2 ± 1.6 | 10.9 ± 0.4 | 10.2 ± 2.7 |
| | 10× | 21.8 ± 3.1 | 63.9 ± 4.3 | 7.5 ± 1.5 | 6.8 ± 0.3 | 9.6 ± 2.8 |
| | 50× | 25.7 ± 2.5 | 55.8 ± 5.8 | 9.4 ± 3.2 | 9.1 ± 1.7 | 8.8 ± 4.4 |
| | 100× | 30.9 ± 6.4 | 48.0 ± 3.0 | 9.2 ± 2.8 | 11.9 ± 2.0 | 6.5 ± 2.1 |
| SKNRA | DMSO | 0.9 ± 0.3 | 65.1 ± 1.8 | 18.4 ± 1.4 | 15.6 ± 0.8 | 3.6 ± 0.4 |
| | 1× | 1.2 ± 0.2 | 86.2 ± 0.7 | 5.6 ± 1.1 | 7.0 ± 0.3 | 16.6 ± 2.9 |
| | 10× | 0.9 ± 0.1 | 76.8 ± 4.7 | 11.4 ± 4.4 | 10.8 ± 0.9 | 8.8 ± 2.8 |
| | 50× | 5.4 ± 2.6 | 74.0 ± 1.5 | 8.7 ± 1.3 | 12.0 ± 0.4 | 8.9 ± 1.3 |
| | 100× | 31.8 ± 5.4 | 40.0 ± 2.4 | 18.0 ± 4.2 | 10.2 ± 0.5 | 2.5 ± 0.7 |
| IMR32 | DMSO | 2.0 ± 0.8 | 56.6 ± 3.2 | 26.9 ± 3.2 | 14.5 ± 0.6 | 2.2 ± 0.3 |
| | 1× | 1.3 ± 0.3 | 58.7 ± 3.5 | 24.9 ± 2.5 | 15.1 ± 0.9 | 2.4 ± 0.4 |
| | 10× | 2.0 ± 0.9 | 66.4 ± 3.9 | 20.4 ± 2.8 | 11.3 ± 2.2 | 3.5 ± 0.7 |
| | 50× | 3.5 ± 2.0 | 63.6 ± 1.5 | 19.8 ± 2.2 | 13.2 ± 1.2 | 3.3 ± 0.5 |
| | 100× | 7.4 ± 2.8 | 62.1 ± 5.9 | 18.7 ± 2.4 | 11.7 ± 1.7 | 3.5 ± 0.7 |
| LAN5 | DMSO | 3.6 ± 0.2 | 47.1 ± 0.9 | 10.6 ± 0.1 | 38.7 ± 1.0 | 2.5 ± 0.1 |
| | 1× | 12.0 ± 1.9 | 37.2 ± 1.2 | 11.0 ± 0.9 | 39.8 ± 1.7 | 4.5 ± 0.5 |
| | 10× | 19.4 ± 5.2 | 31.0 ± 2.5 | 15.1 ± 2.7 | 34.5 ± 5.5 | 3.9 ± 1.1 |
| | 50× | 15.6 ± 8.2 | 31.3 ± 3.8 | 15.0 ± 2.8 | 38.0 ± 7.3 | 2.3 ± 0.7 |
| | 100× | 2.0 ± 7.0 | 56.6 ± 2.8 | 26.9 ± 4.0 | 14.5 ± 8.1 | 2.4 ± 0.7 |
| NGP | DMSO | 0.9 ± 0.0 | 56.1 ± 2.3 | 24.8 ± 2.6 | 18.2 ± 1.5 | 2.3 ± 0.4 |
| | 1× | 1.0 ± 0.2 | 72.2 ± 3.9 | 14.6 ± 1.7 | 12.2 ± 2.41 | 5.1 ± 0.8 |
| | 10× | 2.9 ± 1.7 | 86.5 ± 1.5 | 2.2 ± 0.0 | 8.3 ± 2.6 | 39.0 ± 0.1 |
| | 50× | 4.6 ± 3.3 | 75.2 ± 1.7 | 2.6 ± 0.4 | 17.6 ± 3.5 | 30.5 ± 5.7 |
| | 100× | 3.0 ± 1.1 | 74.8 ± 3.0 | 3.1 ± 0.6 | 19.1 ± 3.2 | 25.9 ± 4.8 |
| NB1691 | DMSO | 0.9 ± 0.1 | 67.2 ± 0.4 | 21.7 ± 0.5 | 10.2 ± 0.1 | 3.1 ± 0.1 |
| | 1× | 1.2 ± 0.2 | 87.1 ± 1.1 | 3.8 ± 0.8 | 7.9 ± 1.3 | 24.8 ± 5.3 |
| | 10× | 3.6 ± 0.5 | 57.8 ± 0.7 | 10.9 ± 2.2 | 27.7 ± 1.0 | 5.7 ± 1.0 |
| | 50× | 6.9 ± 0.6 | 49.5 ± 1.3 | 13.7 ± 1.7 | 30.0 ± 2.3 | 3.7 ± 0.3 |
| | 100× | 5.7 ± 1.5 | 48.6 ± 1.9 | 15.4 ± 1.6 | 30.4 ± 1.9 | 3.2 ± 0.2 |
| Per-108 | DMSO | 0.9 ± 0.1 | 63.8 ± 1.6 | 20.3 ± 1.0 | 15.1 ± 0.6 | 3.2 ± 0.2 |

| | | | | | | |
|---|---|---|---|---|---|---|
| | 1× | 1.4 ± 0.1 | 90.0 ± 0.7 | 4.3 ± 0.3 | 4.3 ± 0.5 | 21.1 ± 1.7 |
| | 10× | 2.5 ± 1.3 | 88.7 ± 2.2 | 3.1 ± 0.6 | 5.7 ± 0.9 | 31.9 ± 8.3 |
| | 50× | 2.9 ± 1.0 | 85.8 ± 1.7 | 3.0 ± 0.1 | 8.3 ± 1.0 | 29.2 ± 1.5 |
| | 100× | 3.0 ± 1.0 | 83.5 ± 1.3 | 2.9 ± 0.1 | 10.6 ± 1.9 | 28.7 ± 1.5 |
| **GIMEN** | DMSO | 1.6 ± 1.2 | 72.3 ± 1.8 | 18.2 ± 3.2 | 7.9 ± 0.2 | 4.3 ± 1.0 |
| | 1× | 1.7 ± 1.0 | 85.2 ± 2.2 | 4.7 ± 2.2 | 8.4 ± 1.5 | 26.6 ± 8.8 |
| | 10× | 2.2 ± 1.1 | 79.4 ± 2.2 | 3.2 ± 0.4 | 15.2 ± 1.6 | 25.3 ± 2.8 |
| | 50× | 2.8 ± 0.7 | 75.2 ± 2.3 | 5.7 ± 2.5 | 16.3 ± 3.4 | 18.0 ± 5.4 |
| | 100× | 6.4 ± 1.7 | 64.4 ± 3.6 | 6.8 ± 1.8 | 22.4 ± 1.7 | 11.0 ± 2.8 |
| **Tet21N MYCN+** | DMSO | 0.7 ± 0.1 | 69.4 ± 4.2 | 16.7 ± 2.7 | 13.2 ± 1.5 | 4.2 ± 0.8 |
| | 1× | 0.8 ± 0.1 | 83.7 ± 4.6 | 7.8 ± 3.2 | 7.7 ± 1.8 | 14.4 ± 8.0 |
| | 10× | 0.8 ± 0.1 | 89.0 ± 1.6 | 3.3 ± 1.2 | 6.9 ± 1.3 | 32.1 ± 10.1 |
| | 50× | 8.9 ± 6.9 | 78.6 ± 8.6 | 4.2 ± 1.5 | 8.3 ± 2.6 | 33.7 ± 14.7 |
| | 100× | 25.6 ± 14.0 | 56.7 ± 19.1 | 11.7 ± 5.0 | 6.0 ± 0.7 | 16.1 ± 9.3 |
| **Tet21N MYCN-** | DMSO | 0.7 ± 0.2 | 86.2 ± 3.4 | 6.2 ± 2.2 | 7.0 ± 1.0 | 17.6 ± 5.3 |
| | 1× | 0.9 ± 0.2 | 87.1 ± 0.4 | 4.3 ± 0.2 | 7.6 ± 0.2 | 20.3 ± 1.0 |
| | 10× | 1.0 ± 0.1 | 89.0 ± 2.4 | 3.5 ± 1.1 | 6.6 ± 1.2 | 30.8 ± 8.0 |
| | 50× | 1.1 ± 0.5 | 85.2 ± 5.6 | 2.0 ± 0.7 | 11.6 ± 4.5 | 52.6 ± 16.4 |
| | 100× | 8.5 ± 6.0 | 75.1 ± 13.0 | 7.1 ± 5.4 | 9.3 ± 1.7 | 36.0 ± 17.0 |
